# Maternal BMI and Diagnostic Accuracy of Using Estimated Fetal Growth to Predict Abnormal Birthweight: Results from NICHD Fetal Growth Studies

**DOI:** 10.3390/diagnostics15111398

**Published:** 2025-05-31

**Authors:** Soutik Ghosal, Jessica L. Gleason, Katherine L. Grantz, Zhen Chen

**Affiliations:** 1 Division of Biostatistics, Public Health Science Department, School of Medicine, University of Virginia, Charlottesville, VA 22904, USA; soutik.ghosal@virginia.edu; 2 Division of Population Health Research, *Eunice Kennedy Shriver* National Institute of Child Health and Human Development, National Institutes of Health, Bethesda, MD 20817, USA; jessica.gleason@nih.gov (J.L.G.); katherine.grantz@nih.gov (K.L.G.)

**Keywords:** biomarkers, abnormal birthweight outcomes, BMI, diagnosis, ROC curve

## Abstract

**Background/Objectives:** The objective of this study was to assess the diagnostic accuracy of sonographic estimated fetal weight (EFW) in predicting small (SGA)- or large-for-gestational-age (LGA) birthweight and examine whether the accuracy is associated with maternal body mass index (BMI). **Methods:** The participants of NICHD Fetal Growth Studies with complete data on maternal BMI (10–13.9 weeks), EFW within 14 days of delivery (18–41.3 weeks), and birthweight were included in this study. Participants were categorized as normal (BMI 18.5–24.9 kg/m^2^) or overweight/obese (BMI > 24.9 to 44.9 kg/m^2^). EFW accuracy was evaluated using area under the Receiver Operating Characteristic curves (AUCs) for SGA and LGA classification, and EFW error was analyzed across BMI groups. **Results:** Among 1289 women, 714 (55.4%) were in the normal BMI group. AUCs for LGA prediction were similar between BMI groups (0.77 ± 0.03 for normal vs. 0.79 ± 0.02 for overweight/obese, *p* = 0.593). However, for SGA, AUCs were higher in the overweight/obese group (.91 ± 0.01 vs. 0.84 ± 0.02, *p* = 0.004), indicating improved accuracy. EFW absolute and percent errors were comparable across BMI groups in the full, AGA, and LGA birth cohorts separately, but they trended lower (*p* = 0.12 and 0.15 for absolute and percent errors, respectively) in the overweight/obese group in the SGA birth cohort. **Conclusions:** EFW has acceptable accuracy for predicting LGA, unaffected by BMI. However, for SGA, EFW accuracy is significantly higher in the overweight/obese group, suggesting that BMI influences diagnostic performance in SGA but not LGA classification.

## 1. Introduction

In obstetrics, abnormal birthweight is an important predictor of complications among both newborns and mothers and an important indicator of neonatal morbidity. Macrosomia or large for gestational age (LGA), defined as a birthweight >the 10th percentile for gestational age (GA), and small for gestational age (SGA), defined as a birthweight <the 10th percentile for GA, are some well-studied birthweight categories used as a proxy for abnormal growth [1,2]. LGA is associated with a range of neonatal complications, including shoulder dystocia, brachial plexus injury, and birth asphyxia [3,4,5], while SGA as a proxy for intrauterine growth restriction is associated with an increased risk of hypoxia, perinatal asphyxia, and long-term developmental delays [6,7]. The delivery of LGA neonates also poses risks for mothers including genital tract injury, prolonged labor, and postpartum bleeding [8,9,10,11,12]. The early prediction of SGA and LGA is clinically important as it improves the management of delivery and postnatal care and reduces neonatal and maternal risks. Estimated fetal weight (EFW) using ultrasound fetal biometry has been studied extensively as such a predictor [13,14,15,16,17].

The accuracy of sonographic EFW in predicting birthweight could potentially be influenced by maternal body mass index (BMI), as ultrasound waves might be attenuated by excessive adipose tissues and abdominal fat, resulting in reduced image quality, difficulty in the visualization of fetal structures, and therefore more inaccurate measurements [18]. The associations of maternal BMI and the accuracy of EFW in predicting birthweight in the literature are mixed. Primarily using the difference between EFW and actual birthweight as a measure of error, studies have shown that maternal BMI does not impact the predictive power of EFW [19,20,21,22,23,24]. Others [25,26] have found that the accuracy of EFW is lower (i.e., the error is higher) for women with higher BMI. Another study also found that high maternal BMI limits the visualization of fetal anatomy during a standard ultrasound examination at 18 to 24 weeks [27].

These studies all assumed that the effects of maternal BMI on EFW accuracy are uniform for the entire distribution of birthweight. It is possible that any potential BMI effect may vary at different parts of the birthweight distribution. The tail regions of birthweight distribution are of special clinical importance, as the lower and upper deciles correspond to SGA and LGA, respectively. Neel et al. [28] evaluated the ability of the third trimester EFW to discriminate SGA and LGA but did not investigate the impact of BMI (they restricted their study to women with BMI > 35 kg/m^2^). They found the limited ability of EFW in identifying SGA and LGA. In comparison, Dude et al. [29] found a higher sensitivity of EFW in discriminating SGA and LGA than Neel study’s did, but the overall diagnostic accuracy measures were not significantly different between women with BMI 35–39 kg/m^2^ and those with a BMI ≥ 40 kg/m^2^. This null finding could have been a result of low statistical power, as the sample size was small [29]. Both studies were in a single hospital setting and included only participants with obese BMI, which may limit the generalizability of the findings. Without a normal BMI group for comparison, it is hard to determine how BMI affects sensitivity, especially since factors like sonographer skill can also influence accuracy.

In this paper, we used data from a large pregnancy cohort to estimate the diagnostic accuracies of late third trimester sonographic EFW in predicting SGA and LGA and to examine whether these diagnostic accuracy measures were associated with maternal BMI. We also investigated the accuracy of EFW in different parts of the birthweight distribution.

## 2. Materials and Methods

The NICHD Fetal Growth Studies-Singletons [30,31,32] included women who were obese (pre-pregnancy BMI: 30–44.9 kg/m^2^) and non-obese (pre-pregnancy BMI: 19–29.9 kg/m^2^). All participants were aged 18–40 years, had a viable singleton pregnancy, and intended to deliver at one of the participating hospitals. Recruitment took place at 12 clinical sites across the U.S. between July 2009 and January 2013, with follow-up through delivery. Human subjects’ approval was obtained from all participating sites, the NICHD, and the data coordinating center, and all women gave written informed consent prior to any data collection (ClinicalTrials.gov Identifier: NCT00912132).

Following a standardized sonogram at 10w0d-13w6d, each woman was randomized to 1 of 4 follow-up visit schedules with 5 additional study sonograms (targeted ranges: 16–22, 24–29, 30–33, 34–37, and 38–41 gestational weeks). Study visits could occur ±1 week from the targeted GA. Study sonographers underwent training and credentialing prior to enrollment and followed a standardized protocol. Ultrasound measurements were performed using standard operating procedures and identical equipment. Fetal biometry included head circumference (HC) and abdominal circumference (AC) using the ellipse function, and femur length (FL) using the linear function was measured at all study visits including 10w0d-13w6d. Voluson ultrasound machines were configured so that the sonographers were blinded to the measurements. EFW was computed from HC, AC, and FL using a formula of Hadlock et al. [33]. Measurements and images were captured in ViewPoint (GE Healthcare) and electronically transferred to this study’s imaging data coordination center. Quality assurance was performed on 5% of the scans and demonstrated correlations between the site sonographers and experts >0.99 for all biometric parameters and coefficients of variation ≤ 3%. In-person interviews were conducted at each research visit to ascertain information on lifestyle and reproductive and medical history. Demographic data; antenatal history; and labor, delivery, and neonatal course and outcomes were abstracted from the prenatal record, labor and delivery summary, and hospital and neonatal records by trained research personnel.

Birthweight distribution was divided into three parts: SGA, where birthweight is below the 10th percentile for GA; LGA, where birthweight is above the 90th percentile for GA; and AGA, between the 10th and 90th percentiles of birthweight [34]. Absolute error was defined as the absolute value of estimated fetal weight minus birthweight, and absolute percent error was defined as absolute error divided by birthweight multiplied by 100. The EFW of each woman at the sonographic visit within 14 days of the delivery date was used. If a woman had multiple ultrasound visits within 14 days of the delivery date, only the last EFW was used.

We compared the demographic and obstetric characteristics of women characterized by maternal BMI group using a *t*-test for continuous variables and a chi-square test for categorical variables. The Lehman family of ROC model [35] was used to estimate the AUC and associated standard errors and 95% confidence intervals. Comparisons were considered statistically significant at the *p* < 0.05 level for two-sided hypotheses. Analyses were performed using R (version 4.0.2, http://www.R-project.org, accessed on 27 April 2025).

Sensitivity analyses were performed across various subsets of the data to examine the robustness of the primary findings. One such analysis relaxed the 14-day threshold of a sonographic visit to within 7, 21, and 28 days of delivery. Additional sensitivity analyses were performed by restricting the delivery time to different GA windows, 34–36, 36–38, and 38–40 weeks, as compared to an unrestricted window in the primary analysis. Further sensitivity analyses were conducted on data with only term pregnancies and on data with only nulliparous women. All the sensitivity analysis results are tabulated in the Supplementary Materials.

## 3. Results

Of the enrolled women in the NICHD Fetal Growth Studies-Singletons, 1289 were used in the analysis; see the flow chart in Supplementary Figure S1 for the various data attritions. Of these 1289 subjects, 99 (7.7%), 1060 (82.2%), and 130 (10.1%) of their newborns were classified as SGA, AGA, and LGA, respectively. The BMI measured at enrollment was used to classify women into the normal (BMI: 18.5–24.9) and overweight/obese (BMI: ≥25 to 45.0) groups. Respectively, 714 (55.4%) and 575 (44.6%) were in the normal and overweight/obese categories. Table 1 presents the baseline characteristics of the cohort stratified by maternal BMI group. While maternal age, GA at ultrasound visit, and GA at delivery were not found to be associated with BMI (*p* = 0.2, >0.9 and =0.8, respectively), race and parity were associated (*p* < 0.001 for both). Overall, average birthweight was significantly higher for the overweight/obese compared to normal BMI group (*p* < 0.001, see also Supplementary Figure S2). For the SGA births, the differences were not found to be statistically significant (*p* > 0.9). In contrast, for LGA births, the average birthweight was higher in the overweight/obese group than in the normal BMI group (*p* = 0.011). Although the proportion of LGA was higher in the overweight/obese BMI group than in the normal group (*p* < 0.001), the proportions of SGA were not different between the two (*p* = 0.13). Overall, the amniotic fluid did not vary between the BMI groups (*p* = 0.4) or SGA/AGA/LGA groups (*p* > 0.9, =0.5, and =0.2, respectively).

The absolute (percent) errors of EFW for the entire cohort (Table 2, top block) ranged from 251.4 ± 181.5 g (7.6 ± 5.4) to 258.6 ± 209.1 g (7.6 ± 5.9) for the normal and overweight/obese BMI groups, respectively, although the differences were not statistically significant (*p* = 0.5 for absolute error and *p* = 0.9 for absolute percent error). The proportion of ultrasound EFW within ±10% and within ±20% of the birthweight was similar between the BMI groups for the entire cohort: the percentages of EFW within ±10% (±20%) were 70.9% (96.5%) and 71.0% (96.9%) for the normal and overweight/obese BMI groups, respectively (*p* > 0.9 and =0.7, respectively).

Insignificant associations between BMI and absolute and percent errors were found separately for the AGA and LGA cohorts. For the AGA cohort, the absolute (percent) errors of EFW ranged from 249.2 ± 178.4 g (7.6 ± 5.4) to 253.0 ± 191.6 g (7.6 ± 5.8) with *p* = 0.7 and 0.9, respectively, for absolute error and absolute percent error (Table 2). For the LGA cohort, the absolute (percent) errors of EFW were 346.4 ± 223.4 g (8.7 ± 5.7) and 348.6 ± 297.8 g (8.4 ± 7.1) with *p* = 0.6 and 0.5 (Table 2). Although the overweight/obese BMI group had a slightly higher absolute error for LGA and AGA, the opposite was observed for SGA. Among the SGA cohort, the absolute (percent) errors of EFW were 191.0 ± 137.5 g (7.2 ± 5.1) and 141.9 ± 92.9 g (5.5 ± 3.8) for the normal and overweight/obese BMI groups, respectively, with *p* = 0. 12 and 0.15 (Table 2). Although not significant, these results suggest that EFW has a higher accuracy in predicting birthweight among women in the overweight/obese BMI group than those in the normal BMI group in the SGA category.

Similar percentages classified within ±10% and ±20% of the true birthweight for both the AGA and LGA cohorts. For the AGA cohort, the percentages of EFW within ±10% (±20%) were 71.3% (96.2%) and 70.7% (96.7%) for the normal and overweight/obese BMI groups, respectively (*p* = 0.8 and 0.6). For the LGA cohort, the percentages of EFW within ±10% (±20%) were 66.0% (96.2%) and 64.9% (96.1%) for the normal and overweight/obese BMI groups, respectively (*p* = 0.9 and >0.9). In contrast, for the SGA cohort, the percentages of EFW within ±10% (±20%) were 71.0% (100%) and 86.5% (100%) for the normal and overweight/obese BMI groups, respectively (*p* = 0.077 for within ±10% and not available for within ±20%), indicating higher accuracy for the overweight/obese group, despite the lack of statistical significance. These findings were also consistent with those based on absolute (percent) errors.

The discriminatory capacity of EFW to differentiate LGA and non-LGA birthweight was not different (*p* = 0.562) between the two BMI groups (AUC 0.769 and 0.788, respectively, for the normal and overweight/obese groups, Table 3; see also Supplementary Figure S3). For differentiating SGA from non-SGA, the discriminating capacity differed between BMI groups (*p* = 0.002) with AUC estimates of 0.839 and 0.911 for the normal and overweight/obese groups, respectively. This confirms the findings using absolute (percent) errors and prediction within ±10% or ±20% of birthweight. For both BMI groups, the AUC estimates for discriminating SGA from non-SGA were found to be higher than those for discriminating LGA from non-LGA.

The sensitivity results using different lengths of time between the last EFW and birthweight (7, 21, 28 days) were generally consistent with the primary findings (Supplementary Tables S1–S3 and Figure S3). Specifically, there were no differences in diagnostic accuracy for LGA between BMI groups, but for discriminating SGA, the diagnostic accuracy estimates of the overweight/obese group were all higher than those for the normal group.

The analyses using EFW from different GA windows (Supplementary Tables S4 and S5, Figure S3) yielded AUC estimates (Supplementary Table S4) that were directionally consistent with the primary findings. For the AGA and LGA cohorts, errors remained stable or showed non-significant increases with higher BMI. However, for the SGA cohort, error metrics were lower in the overweight/obese group within the 38–40 week GA window (*p* = 0.019 and 0.028 for absolute error and absolute percent error, respectively; Supplementary Table S5).

Similarly, analyses restricted to term pregnancies and nulliparous women (Supplementary Tables S6 and S8, Figure S3) showed comparable patterns to the primary results. While the error trends for term pregnancies (Supplementary Table S7) aligned with the main findings, the results for nulliparous women (Supplementary Table S9) diverged. Specifically, for the overall, SGA, and LGA cohorts, errors tended to decrease—though not significantly—with increasing BMI.

## 4. Discussion

The novel findings in this paper contribute to our understanding of the diagnostic capacities of EFW in differentiating between SGA and non-SGA, as well as LGA and non-LGA fetuses. Specifically, we found that when distinguishing between SGA and non-SGA, the diagnostic capacity of EFW was notably higher in the overweight/obese group compared to the normal BMI group. Our sensitivity analyses, including those using different intervals between the last EFW and birthweight (7, 21, and 28 days), produced results that supported our primary conclusion. This finding suggests that maternal BMI may play a role in the accuracy of EFW in predicting SGA in these populations. However, when attempting to discriminate between LGA and non-LGA, the results were less clear-cut. The direction of the estimates for LGA discrimination was mixed, and none of the estimates reached statistical significance. The combination of SGA/higher BMI that improves the predictive power of EFW is notable. SGA fetuses include those with intrauterine growth restriction (IUGR), which is often caused by placental insufficiency or other pathological conditions, such as maternal pregestational diabetes and hypertensive disorders of pregnancy [36]. In women with higher BMI, these factors may be more pronounced, leading to a more apparent discrepancy between the expected and actual fetal growth.

Interestingly, the diagnostic capacity to discriminate SGA from non-SGA was generally higher than that of discriminating LGA from non-LGA. This higher discriminant ability was corroborated by the finding that the absolute (percent) errors of EFW were generally higher for LGA and lower for SGA, which is consistent with what others have found [37]; measurements may be more accurate in a smaller fetus. In the case of LGA, there are often difficulties in accurately measuring fetal dimensions due to the larger size of the fetus. This can introduce measurement errors that are more significant in LGA fetuses compared to SGA fetuses, especially in cases of maternal factors that affect the clarity of ultrasound imaging. Moreover, birthweight was significantly higher in the overweight/obese BMI group compared to the normal BMI group within the LGA cohort, but no such difference was observed in the SGA cohort (Table 1 and Supplementary Figure S2). This between-group variability likely introduces more heterogeneity into the LGA cohort, which in turn may diminish the diagnostic accuracy of EFW for identifying LGA fetuses.

The unexpected finding of higher discriminant ability for SGA in women with overweight or obesity compared to women with normal BMI warrants further investigation. Although the AUC for predicting SGA was relatively high for both BMI groups, the lower variability in EFW for women with overweight/obese BMI may have contributed to the higher AUC in this group. There may also be unknown factors that influence both maternal BMI and SGA prediction. On the other hand, predicting LGA from EFW is generally challenging, as fetal parameters become more difficult to measure in larger fetuses, particularly toward the end of pregnancy, when the fetal head begins to descend in the pelvis. It has been previously demonstrated that there is generally a higher error in LGA prediction relative to appropriately grown or SGA fetuses [37]. Thus, it is not surprising that LGA prediction is unaffected by maternal BMI and prediction accuracy is poor across BMI groups.

There are a few limitations in this study that should be considered when interpreting the results. Firstly, the study cohort predominantly consisted of women with lower-risk pregnancies, which may limit the generalizability of the findings to higher-risk populations. Women with complex medical conditions or those who are at a higher risk for adverse pregnancy outcomes may exhibit different diagnostic patterns, and the findings from this cohort may not fully apply to these groups.

Another limitation lies in the BMI categorization. The study groups—normal BMI and overweight/obese—were based on the maternal BMI measured at the beginning of the pregnancy rather than on the BMI closer to delivery. Maternal weight and BMI can change significantly throughout pregnancy, and these changes may influence fetal growth and the diagnostic accuracy of EFW. By relying solely on baseline BMI, this study may not fully account for these changes, potentially affecting the accuracy of its findings.

## Figures and Tables

**Table 1:** Characteristics of study participants by BMI group, 14-day threshold. Continuous characteristics are presented as Mean ± SD and compared between BMI groups using nonparametric Wilcoxon rank sum test; categorical characteristics are presented as *n* (%) and compared between BMI groups using chi-square test (* denotes significant difference between BMI groups).

Characteristics	BMI Group		*p*
	Normal	Overweight/Obese	
	(*n* = 714)	(*n* = 575)	
Age (years)	28.6 ± 5.4	28.3 ± 5.5	0.2
Race			<0.001 *
White	221 (31)	154 (26.8)	
African American	155 (21.7)	189 (32.9)	
Hispanic	172 (24.1)	190 (33)	
Asian and Pacific Islander	166 (23.2)	42 (7.3)	
BMI (kg/m^2^) at enrollment	21.9 ± 1.7	30.0 ± 4.6	<0.001 *
Parity			<0.001 *
Parity: Nulliparous	359 (50.3)	212 (36.9)	
Parity = 1	252 (35.3)	210 (36.5)	
Parity > 1	103 (14.4)	153 (26.6)	
Amniotic fluid index	14.1 ± 4.7	14.4 ± 4.8	0.4
GA at US visit (weeks)	38.2 ± 1.5	38.2 ± 1.6	>0.9
GA at delivery (weeks)	39.3 ± 1.4	39.3 ± 1.6	0.8
Time to delivery (days)	7.8 ± 4.1	7.6 ± 4.0	0.4
Birthweight (g)	3312.6 ± 461.1	3434.4 ± 496	<0.001 *
EFW (g)	3182.1 ± 504.4	3309.7 ± 547.3	<0.001 *
SGA	62 (8.7)	37 (6.4)	0.13
LGA	53 (7.4)	77 (13.4)	0.001 *

**Table 2:** EFW prediction results by BMI group, for different birthweight categories, 14-day threshold. Continuous characteristics are presented as Mean ± SD and compared between BMI groups using nonparametric Wilcoxon rank sum test; categorical characteristics are presented as *n* (%) and compared between BMI groups using chi-square test (* denotes significant difference between BMI groups).

Birthweight Category	Characteristics	BMI Group		*p*
		Normal	Overweight/Obese	
All	*n*	714	575	
	EFW within 10% of birthweight	506 (70.9)	408 (71.0)	>0.9
	EFW within 20% of birthweight	689 (96.5)	557 (96.9)	0.7
	Absolute error (g)	251.4 ± 181.5	258.6 ± 209.1	0.9
	Absolute percent error	7.6 ± 5.4	7.6 ± 5.9	0.5
SGA	*n*	62	37	
	EFW within 10% of birthweight	44 (71.0)	32 (86.5)	0.077
	EFW within 20% of birthweight	62 (100.0)	37 (100.0)	-
	GA at US visit (weeks)	38.0 ± 1.2	38.1 ± 1.4	0.6
	GA at delivery (weeks)	39.2 ± 1.3	39.1 ± 1.4	0.9
	Time to delivery (days)	8.3 ± 4.3	7.5 ± 3.6	0.3
	EFW (g)	2617.2 ± 394.4	2604 ± 295.8	>0.9
	Amniotic fluid index	11.9 ± 4	11.8 ± 3.9	>0.9
	Birthweight (g)	2648.1 ± 267.2	2659.8 ± 241.4	>0.9
	Absolute error (g)	191.0 ± 137.5	141.9 ± 92.9	0.12
	Absolute percent error	7.2 ± 5.1	5.5 ± 3.8	0.15
AGA	*n*	599	461	
	EFW within 10% of birthweight	427 (71.3)	326 (70.7)	0.8
	EFW within 20% of birthweight	576 (96.2)	446 (96.7)	0.6
	GA at US visit (weeks)	38.2 ± 1.5	38.2 ± 1.7	0.8
	GA at delivery (weeks)	39.3 ± 1.4	39.3 ± 1.6	0.6
	Time to delivery (days)	7.8 ± 4.0	7.6 ± 4.0	0.5
	EFW (g)	3188.3 ± 452.8	3262.4 ± 470.4	0.016
	Amniotic fluid index	14.1 ± 4.7	14.3 ± 4.7	0.5
	Birthweight (g)	3317 ± 380.8	3376 ± 384.3	0.009 *
	Absolute error (g)	249.2 ± 178.4	253.0 ± 191.6	0.9
	Absolute percent error	7.6 ± 5.4	7.6 ± 5.8	0.7
LGA	*n*	53	77	
	EFW within 10% of birthweight	35 (66.0)	50 (64.9)	0.9
	EFW within 20% of birthweight	51 (96.2)	74 (96.1)	>0.9
	GA at US visit (weeks)	38.1 ± 1.3	38.2 ± 1.2	0.6
	GA at delivery (weeks)	39.2 ± 1.2	39.3 ± 1.1	0.3
	Time to delivery (days)	7.8 ± 3.9	8 ± 4.2	0.8
	EFW (g)	3772.5 ± 460.7	3931.4 ± 483.6	0.03 *
	Amniotic fluid index	17.0 ± 4.8	16.0 ± 5.2	0.2
	Birthweight (g)	4040.7 ± 310.7	4156.7 ± 309.1	0.011 *
	Absolute error (g)	346.4 ± 223.4	348.6 ± 297.8	0.6
	Absolute percent error	8.7 ± 5.7	8.4 ± 7.1	0.5

**Table 3:** AUC estimates of EFW for discriminating LGA and SGA by BMI group, 14-day threshold (* denotes significant difference between BMI groups based on Wald test).

BMI Group	SGA			LGA		
	Est	SE	*p*	Est	SE	*p*
Normal	0.839	0.019	0.002 *	0.769	0.027	0.562
Overweight/Obese	0.911	0.015		0.788	0.022	

## Data Availability

The data generated by this project will be available upon reasonable request from the corresponding author.

## Supplementary Materials

The following supporting information can be downloaded at https://www.mdpi.com/article/10.3390/diagnostics15111398/s1. In the Supplementary Material, we mainly provide sensitivity analyses to validate our main findings. Sensitivity analyses were performed for different subsets of the data categorized by different thresholds (Supplementary Tables S1–S3), different GA windows (Supplementary Tables S4 and S5), only term pregnancy at the 14-day threshold (Supplementary Tables S6 and S7), and only the nulliparous cohort at the 14-day threshold (Supplementary Tables S8 and S9).

## Abbreviations

The following abbreviations are used in this manuscript:

SGA	Small for gestational age
LGA	Large for gestational age
AGA	Appropriate for gestational age
GA	Gestational age
BMI	Body mass index
EFW	Estimated fetal weight
NICHD	*Eunice Kennedy Shriver* National Institute of Child Health & Human Development
ROC	Receiver Operating Characteristic
AUC	Area under the ROC curve
HC	Head circumference
FL	Femur length
AC	Abdominal circumference
