# Supplementary material for: Maternal BMI and Diagnostic Accuracy of Using Estimated Fetal Growth to Predict Abnormal Birthweight: Results from NICHD Fetal Growth Studies
Source: Diagnostics (Basel). 2025 May 31;15(11):1398. doi: 10.3390/diagnostics15111398 (PMC12155272; doi:10.3390/diagnostics15111398)
Supplement: Supplementary file 1 [file diagnostics-15-01398-s001.zip › diagnostics-3642798-supplementary.pdf]

Title: Supplementary material for Maternal BMI and diagnostic accuracy of estimated fetal growth to predict abnormal birthweight: results from the NICHD Fetal Growth Studies

Authors: Soutik Ghosal, PhD [a], Jessica L. Gleason, PhD, MPH [b], Katherine L. Grantz, MD [b], MS, Zhen Chen, PhD [b]

Affiliation: [a] Division of Biostatistics, Public Health Science Department, School of Medicine, University of Virginia, Charlottesville, VA.

[b] Division of Population Health Research, *Eunice Kennedy Shriver* National Institute of Child Health and Human Development, National Institutes of Health, Bethesda, MD.

Corresponding Author: Zhen Chen, PhD. Division of Population Health Research, *Eunice Kennedy Shriver* National Institute of Child Health and Human Development, Bethesda, MD, United States, zhen.chen@nih.gov.

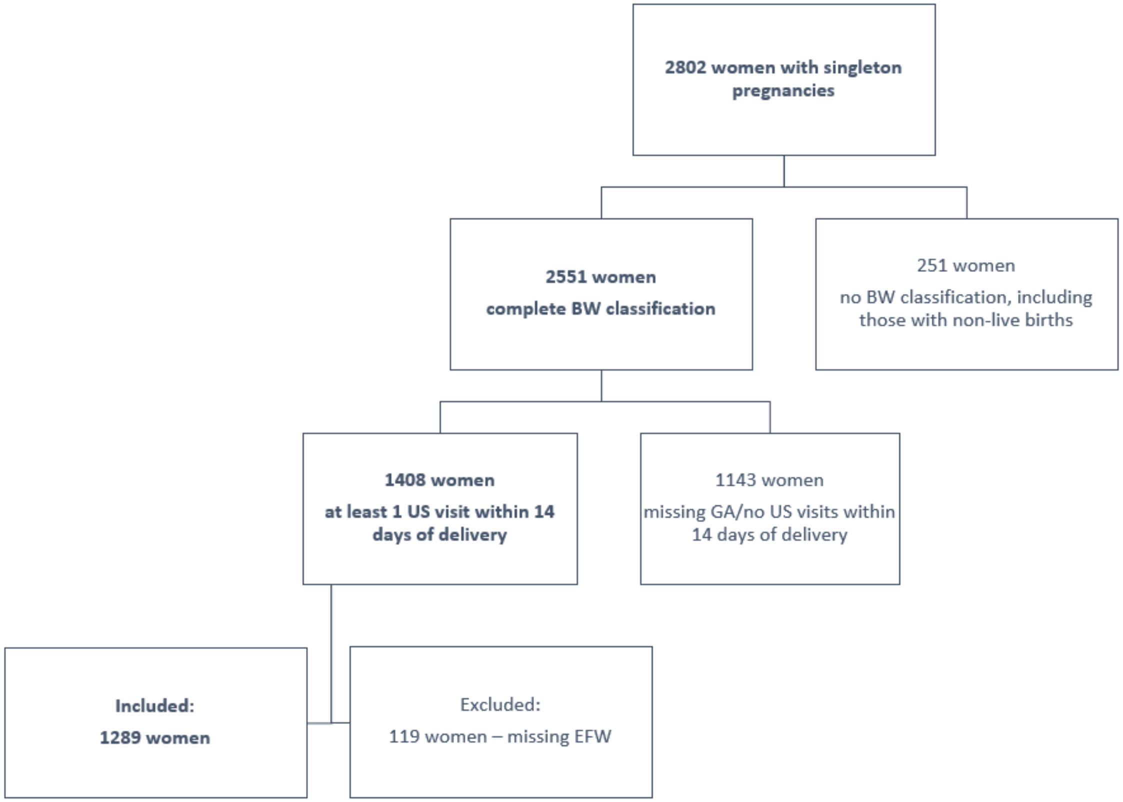

Supp. Figure S1: Flow chart showing analytical data.

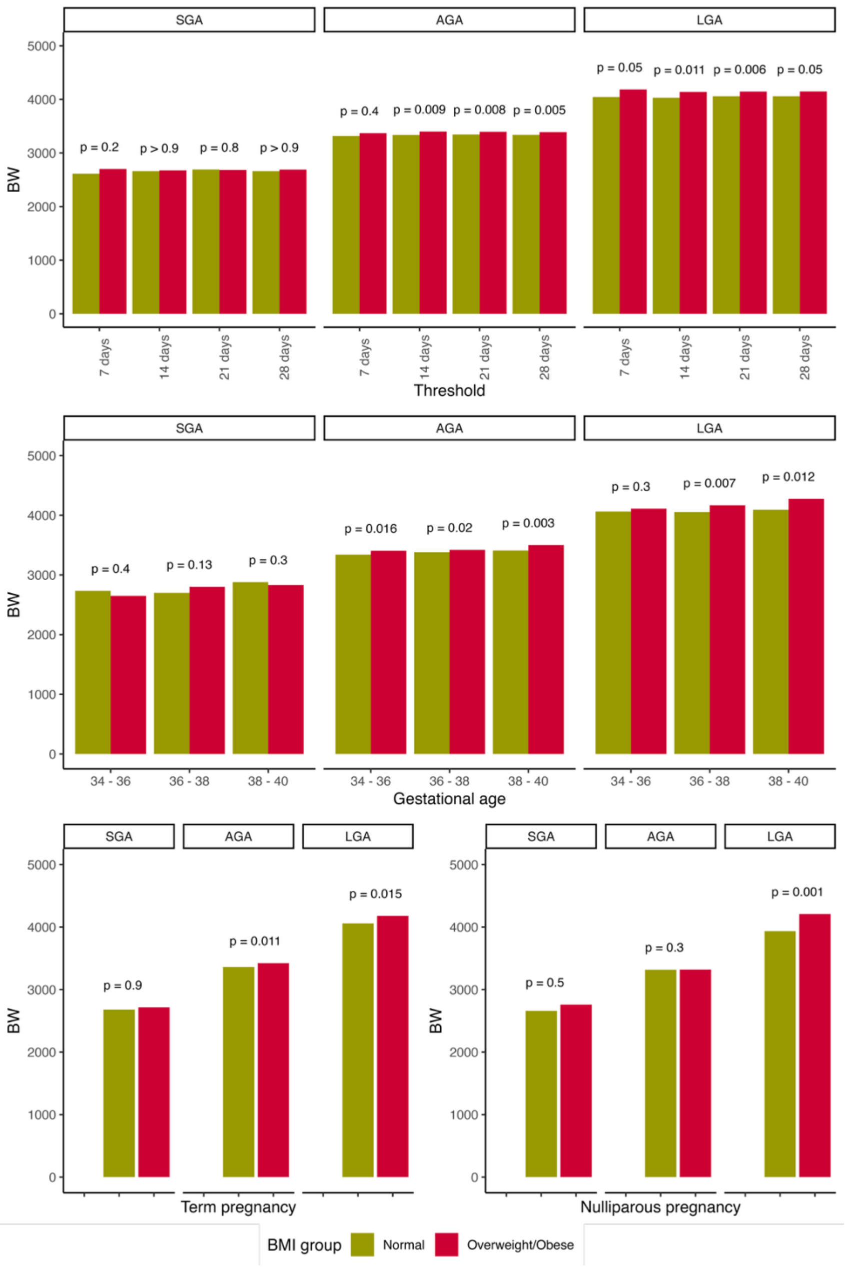

Supp. Figure S2: Birth weight distributions stratified by various factors (p values are based on the nonparametric Wilcoxon rank sum test).

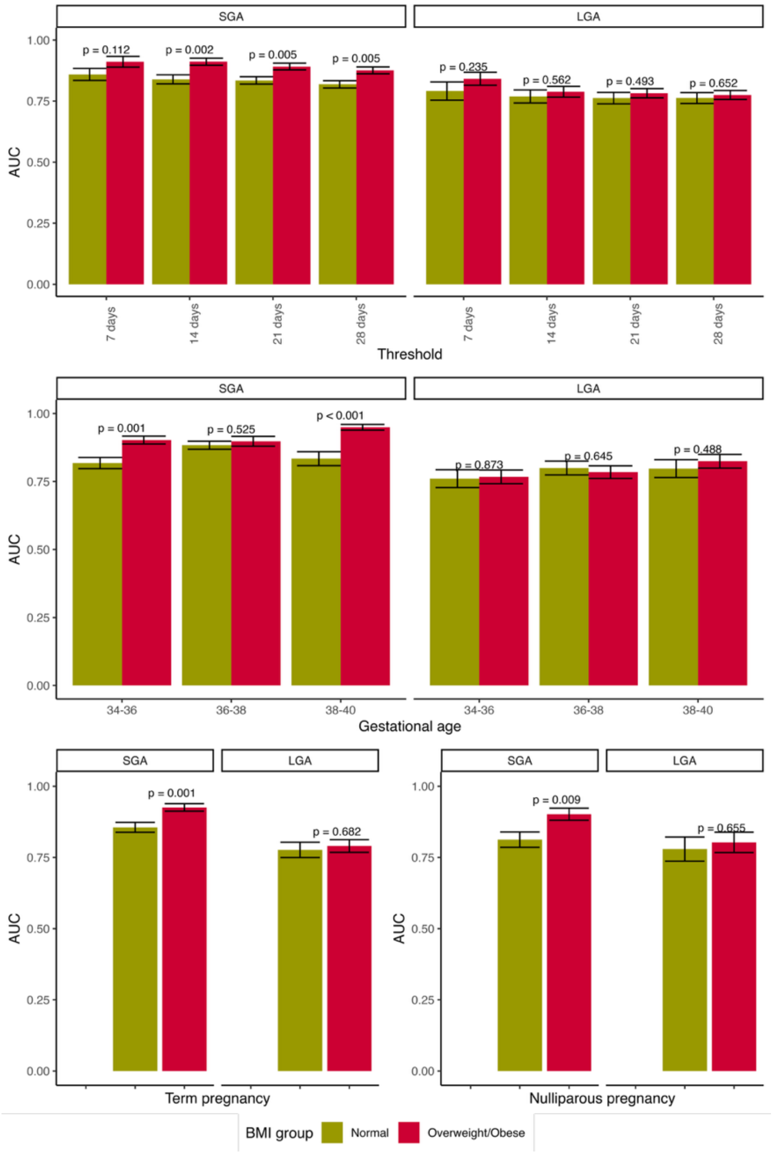

Supp. Figure S3: AUC estimates of EFW for discriminating LGA and SGA by BMI groups (p values are based on the Wald test).

Supp. Table S1. Characteristics of study participants by BMI group, all thresholds. Continuous characteristics are presented as Mean±SD and compared between BMI groups using the nonparametric Wilcoxon rank sum test, categorical characteristics are presented as n (%) and compared between BMI groups using the Chi-square test (denotes significant difference between BMI groups).*

Characteristics	7 days			14 days			21 days			28 days		
	BMI group			BMI group			BMI group			BMI group		
	Normal (n=327)	Overweight/Obese (n=287)	*p*	Normal (n=714)	Overweight/Obese (n=575)	*p*	Normal (n=1033)	Overweight/Obese (n=816)	*p*	Normal (n=1201)	Overweight/Obese (n=956)	*p*
Age (years)	28.5±5.5	28.7±5.5	0.9	28.6±5.4	28.3±5.5	0.2	28.5±5.4	28.2±5.6	0.13	28.4±5.4	28.1±5.6	0.12
Race			<0.001*			<0.001*			<0.001*			<0.001*
Race: White	109 (33.3%)	76 (26.5%)		221 (31%)	154 (26.8%)		311 (30.1%)	224 (27.5%)		353 (29.4%)	261 (27.3%)	
Race: African American	68 (20.8%)	87 (30.3%)		155 (21.7%)	189 (32.9%)		224 (21.7%)	262 (32.1%)		278 (23.1%)	309 (32.3%)	
Race: Hispanic	77 (23.5%)	100 (34.8%)		172 (24.1%)	190 (33.0%)		261 (25.3%)	270 (33.1%)		296 (24.6%)	318 (33.3%)	
Race: Asian & Pacific Islander	73 (22.3%)	24 (8.4%)		166 (23.2%)	42 (7.3%)		237 (22.9%)	60 (7.4%)		274 (22.8%)	68 (7.1%)	
BMI (kg/m^2) at baseline	22.0±1.7	30.0±4.5	<0.001*	21.9±1.7	30.0±4.6	<0.001*	21.9±1.7	29.9±4.5	<0.001*	21.9±1.7	29.9±4.5	<0.001*
Parity			<0.001*			<0.001*			<0.001*			<0.001*
Parity: Nulliparous	162 (49.5%)	93 (32.4%)		359 (50.3%)	212 (36.9%)		530 (51.3%)	311 (38.1%)		626 (52.1%)	376 (39.3%)	
Parity = 1	118 (36.1%)	110 (38.3%)		252 (35.3%)	210 (36.5%)		356 (34.5%)	304 (37.3%)		402 (33.5%)	341 (35.7%)	
Parity > 1	47 (14.4%)	84 (29.3%)		103 (14.4%)	153 (26.6%)		147 (14.2%)	201 (24.6%)		173 (14.4%)	239 (25%)	
Amniotic fluid index	13.8±4.8	14.2±5.1	0.6	14.1±4.7	14.4±4.8	0.4	14.2±4.5	14.5±4.7	0.5	14.2±4.4	14.5±4.6	0.3
GA at US visit (weeks)	38.5±1.4	38.6±1.2	0.5	38.2±1.5	38.2±1.6	>0.9	37.8±1.6	37.7±1.8	0.8	37.5±1.9	37.4±2.2	>0.9
GA at delivery (weeks)	39.1±1.3	39.2±1.1	0.3	39.3±1.4	39.3±1.6	0.8	39.3±1.5	39.3±1.6	0.6	39.3±1.6	39.2±1.9	0.7
Time to delivery (days)	4.0±2.1	4.2±2.1	0.4	7.8±4.1	7.6±4.0	0.4	10.9±5.8	10.6±5.8	0.3	12.8±7.3	12.7±7.4	0.6
Birthweight (g)	3303.2±477.1	3423.4±457.2	0.021*	3312.6±461.1	3434.4±496.0	<0.001*	3312.1±461.4	3417.1±509.4	<0.001*	3300.2±472.7	3399.4±539.8	<0.001*
EFW (g)	3280.5±521.8	3388.9±513.2	0.13	3182.1±504.4	3309.7±547.3	<0.001*	3101.5±510.5	3201.2±564.1	<0.001*	3034.7±532.8	3126.6±600.2	<0.001*
SGA	29 (8.9%)	16 (5.6%)	0.159	62 (8.7%)	37 (6.4%)	0.13	96 (9.3%)	58 (7.1%)	0.109	114 (9.5%)	71 (7.4%)	0.104
LGA	24 (7.3%)	37 (12.9%)	0.031*	53 (7.4%)	77 (13.4%)	0.001*	71 (6.9%)	103 (12.6%)	<0.001*	79 (6.6%)	116 (12.1%)	<0.001*

Supp. Table S2. AUC estimates of EFW for discriminating LGA and SGA by BMI group, all thresholds (denotes significant difference between BMI groups based on the Wald test).*

Threshold	BMI group	SGA			LGA		
		Est	SE	*p*	Est	SE	*p*
7 days	Normal	0.859	0.024	0.112	0.791	0.037	0.235
	Overweight/Obese	0.911	0.022		0.842	0.027	
14 days	Normal	0.839	0.019	0.002*	0.769	0.027	0.562
	Overweight/Obese	0.911	0.015		0.788	0.022	
21 days	Normal	0.835	0.015	0.005*	0.762	0.023	0.493
	Overweight/Obese	0.892	0.014		0.782	0.019	

28 days	Normal	0.819	0.015	0.005*	0.763	0.022	0.652
	Overweight/Obese	0.876	0.014		0.775	0.018	

Supp. Table S3. EFW Prediction results by BMI group, all thresholds. Continuous characteristics are presented as Mean±SD and compared between BMI groups using the nonparametric Wilcoxon rank sum test, categorical characteristics are presented as n (%) and compared between BMI groups using the Chi-square test (denotes significantly difference between BMI groups).*

Birthweight category	Characteristics	7 days			14 days			21 days			28 days		
		BMI group		*p*	BMI group		*p*	BMI group		*p*	BMI group		*p*
		Normal	Overweight/Obese		Normal	Overweight/Obese		Normal	Overweight/Obese		Normal	Overweight/Obese	
All	n	327	287		714	575		1033	816		1201	956	
	EFW within 10% of birth weight	267 (81.7)	224 (78.0)	0.3	506 (70.9)	408 (71.0%)	>0.9	630 (61.0)	490 (60.0)	0.7	652 (54.3)	514 (53.8)	0.8
	EFW within 20% of birth weight	320 (97.9)	283 (98.6)	0.5	689 (96.5)	557 (96.9%)	0.7	952 (92.2)	754 (92.4)	0.8	1040 (86.6)	834 (87.2)	0.7
	Absolute error (g)	208±158.8	219.3±173.6	0.6	251.4±181.5	258.6±209.1	0.9	300.4±215.7	315.3±237	0.4	344.2±248.3	357.6±268.1	0.5
	Absolute percent error	6.4±4.9	6.4±5.0	>0.9	7.6±5.4	7.6±5.9	0.5	9.1±6.4	9.3±6.7	0.8	10.5±7.5	10.7±7.9	>0.9
SGA	n	29	16		62	37		96	58		114	71	
	EFW within 10% of birth weight	20 (69.0)	14 (87.5)	0.3	44 (71.0)	32 (86.5)	0.077	59 (61.5)	42 (72.4)	0.2	63 (55.3)	45 (63.4)	0.3
	EFW within 20% of birth weight	29 (100.0)	16 (100.0)	-	62 (100.0)	37 (100.0)	-	90 (93.8)	58 (100.0)	0.084	102 (89.5)	66 (93.0)	0.4
	Birthweight (g)	2581.3±306.2	2705.8±194.8	0.2	2648.1±267.2	2659.8±241.4	>0.9	2651.3±276.3	2642.3±277.3	0.8	2631.9±281.2	2640.3±277.9	>0.9
	GA at US visit (weeks)	38.2±1.3	38.7±0.7	0.3	38.0±1.2	38.1±1.4	0.6	37.6±1.4	37.5±1.7	0.8	37.3±1.6	37.2±1.8	>0.9
	GA at delivery (weeks)	38.8±1.3	39.3±0.8	0.4	39.2±1.3	39.1±1.4	0.9	39.3±1.3	39.1±1.5	0.8	39.2±1.3	39.1±1.5	>0.9
	Time to delivery (days)	4.3±2.5	4.1±2.3	0.6	8.3±4.3	7.5±3.6	0.3	11.6±5.8	11.2±5.9	0.7	13.6±7.1	13.6±7.5	>0.9
	Amniotic fluid index	10.6±3.0	11.6±4.5	0.2	11.9±4.0	11.8±3.9	>0.9	12.7±4.0	12.3±3.9	0.7	12.6±4.0	12.3±3.6	0.8
	EFW (g)	2606.4±467.8	2698.6±253.2	0.6	2617.2±394.4	2604.0±295.8	>0.9	2534.0±395.8	2521.7±346.5	0.9	2484.6±399.3	2466.1±362.2	>0.9
	Absolute error (g)	198.5±139.6	138.8±96.2	0.2	191.0±137.5	141.9±92.9	0.12	232.9±170.0	175.5±121.7	0.06	259.7±185.4	219.1±172.1	0.11
	Absolute percent error	7.6±5.3	5.3±3.9	0.2	7.2±5.1	5.5±3.8	0.15	8.8±6.3	6.9±4.9	0.09	9.9±7.2	8.5±6.6	0.2
AGA	n	274	234		599	461		866	655		1008	769	
	EFW within 10% of birth weight	227 (82.8)	179 (76.5)	0.075	427 (71.3)	326 (70.7)	0.8	530 (61.2)	390 (59.5)	0.5	547 (54.3)	410 (53.3)	0.7
	EFW within 20% of birth weight	268 (97.8)	231 (98.7)	0.5	576 (96.2)	446 (96.7)	0.6	801 (92.5)	604 (92.2)	0.8	873 (86.6)	673 (87.5)	0.6
	Birthweight (g)	3313.6±389.6	3358.2±334.3	0.4	3317.0±380.8	3376±384.3	0.009*	3324.9±382.1	3369.6±397.2	0.008*	3316.9±395.4	3354.3±441.7	0.005*
	GA at US visit (weeks)	38.6±1.4	38.6±1.2	0.7	38.2±1.5	38.2±1.7	0.8	37.8±1.7	37.7±1.9	>0.90	37.5±2.0	37.4±2.3	0.8
	GA at delivery (weeks)	39.1±1.4	39.2±1.2	0.5	39.3±1.4	39.3±1.6	0.6	39.3±1.5	39.3±1.7	0.5	39.3±1.7	39.2±2	0.6
	Time to delivery (days)	4.0±2.1	4.2±2.1	0.3	7.8±4.0	7.6±4.0	0.5	10.9±5.8	10.6±5.8	0.4	12.8±7.3	12.7±7.5	0.7
	Amniotic fluid index	13.9±4.7	14.0±4.9	0.9	14.1±4.7	14.3±4.7	0.5	14.2±4.5	14.4±4.7	0.7	14.2±4.4	14.4±4.5	0.5
	EFW (g)	3295.8±450.9	3321.3±405.3	>0.9	3188.3±452.8	3262.4±470.4	0.016*	3116.2±459.0	3162.2±490.9	0.057	3048.3±487.7	3091.4±539.5	0.023*
	Absolute error (g)	205.8±156.8	219.9±171.8	0.5	249.2±178.4	253.0±191.6	0.9	298.2±209.3	310.9±222.6	0.4	345.5±246.7	351.1±251.1	0.7
	Absolute percent error	6.3±4.8	6.6±5	0.5	7.6±5.4	7.6±5.8	0.7	9.0±6.3	9.3±6.6	0.5	10.5±7.5	10.7±7.9	0.8
LGA	n	24	37		53	77		71	103		79	116	
	EFW within 10% of birth weight	20 (83.3)	31 (83.8)	>0.9	35 (66.0)	50 (64.9)	0.9	41 (57.7)	58 (56.3)	0.9	42 (53.2)	59 (50.9)	0.8
	EFW within 20% of birth weight	23 (95.8)	36 (97.3)	>0.9	51 (96.2)	74 (96.1)	>0.9	61 (85.9)	92 (89.3)	0.5	65 (82.3)	95 (81.9)	>0.9
	Birthweight (g)	4056.2±246.3	4145.5±358.8	0.005*	4040.7±310.7	4156.7±309.1	0.011*	4048.7±281.1	4156.1±333.9	0.006*	4052.5±274.6	4162.7±335.8	0.005*
	GA at US visit (weeks)	38.5±1.0	38.5±1.3	0.8	38.1±1.3	38.2±1.2	0.6	37.8±1.3	37.8±1.4	0.4	37.6±1.3	37.6±1.5	0.6
	GA at delivery (weeks)	39.1±0.9	39.1±1.2	0.6	39.2±1.2	39.3±1.1	0.3	39.3±1.1	39.3±1.2	0.4	39.3±1.1	39.4±1.2	0.5

Time to delivery (days)	4.2±2.0	4.2±2.1	>0.9	7.8±3.9	8.0±4.2	0.8	10.4±5.7	10.6±5.8	0.9	11.8±6.9	12.2±7.1	0.8
Amniotic fluid index	17.1±4.9	16.6±5.9	0.6	17.0±4.8	16.0±5.2	0.2	16.5±4.9	16.2±5.1	0.6	16.3±4.9	16.1±5.1	0.8
EFW (g)	3920.0±420.1	4115.0±468.0	0.055	3772.5±460.7	3931.4±483.6	0.031*	3688.5±481.2	3831.8±506.7	0.049*	3654.3±480.8	3764.3±529.0	0.1
Absolute error (g)	245.3±200.9	250.4±201.9	>0.9	346.4±223.4	348.6±297.8	0.6	418.6±292.2	422.1±316.1	>0.9	450.7±302.1	485.2±359.7	0.7
Absolute percent error	6.1±5.0	6.1±4.9	>0.9	8.7±5.7	8.4±7.1	0.5	10.4±7.4	10.2±7.6	0.8	11.2±7.6	11.7±8.5	0.9

Supp. Table S4. AUC table stratified by GA (denotes significantly difference between BMI groups based on the Wald test).*

GA	BMI group	SGA			LGA		
		Est	SE	*p*	Est	SE	*p*
34-36	Normal	0.818	0.020	0.001*	0.761	0.033	0.873
	Overweight/Obese	0.902	0.014		0.767	0.025	
36-38	Normal	0.883	0.015	0.525	0.800	0.025	0.645
	Overweight/Obese	0.898	0.018		0.784	0.023	
38-40	Normal	0.834	0.026	< 0.001*	0.797	0.033	0.488
	Overweight/Obese	0.949	0.011		0.824	0.025	

Supp. Table S5. EFW prediction results by BMI group, stratified by GA, for different birth weight categories. *Continuous characteristics are presented as Mean±SD and compared between BMI groups using the nonparametric Wilcoxon rank sum test, categorical characteristics are presented as n (%) and compared between BMI groups using the Chi-square test* *(* denotes significantly difference between BMI groups).*

Birthweight category	Characteristics	34-36 weeks			36-38 weeks			38-40 weeks		
		BMI		*p*	BMI		*p*	BMI		*p*
		Normal	Overweight/Obese		Normal	Overweight/Obese		Normal	Overweight/Obese	
SGA	n	64	45		61	30		34	27	
	EFW within 10% of birth weight	14 (21.9)	8 (17.8)	0.6	28 (45.9)	13 (43.3)	0.8	25 (73.5)	25 (92.6)	0.092
	EFW within 20% of birth weight	36 (56.2)	22 (48.9)	0.4	52 (85.2)	25 (83.3)	>0.9	34 (100.0)	27 (100.0)	-
	Birthweight (g)	2648.1±313.4	2614.0±283	0.4	2671±234.3	2753.6±206.9	0.13	2809.9±174.5	2779.7±161.5	0.3
	GA at US visit (weeks)	35.1±0.6	35.0±0.6	0.6	37.2±0.6	37.1±0.6	0.7	39.0±0.6	38.9±0.5	0.4
	GA at delivery (weeks)	39.3±1.4	39.0±1.5	0.4	39.4±1.1	39.8±1.2	0.11	40.3±0.7	40.0±0.8	0.3
	Time to delivery (days)	29.5±10.7	27.9±10.8	0.4	15.9±8.1	18.6±8.6	0.13	8.9±4.7	8.1±4.8	0.4
	Amniotic fluid index	13.1±3.5	13.8±3.2	0.2	12.8±4.2	12.3±2.3	0.7	11.6±3.1	11.3±4.2	0.9
	EFW (g)	2157.8±266.1	2096.4±233.7	0.3	2444.3±283.9	2468.2±273.8	0.6	2844.8±314.7	2717.7±208.1	0.054
	Absolute error (g)	497.1±282.0	533.0±283.0	0.5	302.2±218.7	317.3±205.2	0.8	195.2±132.1	124.0±87.1	0.035*
	Absolute percent error	18.2±9.5	19.8±9.7	0.4	11.3±8.3	11.5±7.4	0.8	6.9±4.6	4.5±3.3	0.056
AGA	n	521	430		553	408		405	304	
	EFW within 10% of birth weight	34 (6.5)	40 (9.3)	0.11	224 (40.5)	171 (41.9)	0.7	301 (73.1)	219 (71.4)	0.6
	EFW within 20% of birth weight	162 (31.1)	162 (37.7)	0.033	453 (81.9)	326 (79.9)	0.4	397 (96.8)	296 (96.7)	>0.9
	Birthweight (g)	3345.1±331.5	3401.6±346.6	0.016*	3381.2±29.70	3423.6±317.4	0.02*	3433.4±285.5	3495.1±296.2	0.003*
	GA at US visit (weeks)	35.1±0.6	35.1±0.6	0.084	37.1±0.6	37.1±0.6	>0.9	38.9±0.5	38.9±0.5	0.3
	GA at delivery (weeks)	39.4±1.2	39.4±1.2	0.7	39.6±1.0	39.6±1.1	0.8	40.1±0.8	40.1±0.8	>0.9
	Time to delivery (days)	30.7±9.3	30.2±9.5	0.50	17.6±7.8	17.3±8.1	0.6	8.5±5.2	8.2±5.1	0.6
	Amniotic fluid index	14.8±3.9	15.1±4.2	0.4	14.2±4.0	14.9±4.8	0.085	14.1±4.8	14.1±4.4	0.8
	EFW (g)	2540.3±314.0	2606.6±300.8	<0.001*	2980.1±348.8	3018.1±367.4	0.074	3309.0±365.4	3382.6±361.2	0.01*
	Absolute error (g)	807.8±329.7	798.6±356.9	0.6	434.1±272.7	446.4±293.1	0.7	256.2±189.4	258.7±200.8	>0.9
	Absolute percent error	23.8±8.8	23.0±9.3	0.2	12.7±7.6	12.8±8.2	0.8	7.4±5.4	7.4±5.6	0.8
LGA	n	33	65		49	67		30	45	
	EFW within 10% of birth weight	1 (3.0)	4 (6.2)	0.7	20 (40.8)	24 (35.8)	0.6	22 (73.3)	31 (66.7)	0.6
	EFW within 20% of birth weight	8 (24.2)	14 (21.5)	0.8	37 (75.5)	50 (74.6)	>0.9	30 (100.0)	45 (97.8)	>0.9
	Birthweight (g)	4091.8±295.6	4154.8±345.1	0.3	4061.2±210.1	4196.2±271.0	0.007*	4160.9±252.7	4302.9±262.0	0.012*

GA at US visit (weeks)	35.1±0.7	35.1±0.6	0.6	37.1±0.6	37.2±0.6	0.5	38.9±0.5	38.8±0.4	0.4
GA at delivery (weeks)	39.5±1.1	39.4±1.1	0.6	39.3±0.9	39.6±0.8	0.11	40.0±0.8	39.9±0.7	0.6
Time to delivery (days)	30.4±9.0	30.1±9.5	0.9	15.1±6.8	16.7±7.1	0.3	7.4±5.4	7.8±5.5	>0.9
Amniotic fluid index	15.6±3.7	17.4±3.8	0.039*	16.2±5.4	16.1±4.6	0.9	16.5±5.0	16.9±5.4	>0.9
EFW (g)	3010.4±266.6	3057.2±316.6	0.5	3571.4±387.5	3610.8±399.6	0.3	3926.2±370.2	4119.7±368.8	0.023*
Absolute error (g)	1081.4±349.0	1103.8±408.6	0.7	533.4±309.5	610.5±394.0	0.5	301.7±207.5	351.9±233.1	0.4
Absolute percent error	26.2±7.4	26.2±8.9	>0.9	13.1±7.5	14.3±9.0	0.6	7.3±4.9	8.2±5.3	0.6

Supp. Table S6. AUC estimates for term pregnancy, 14 days threshold (denotes significantly difference between BMI groups based on the Wald test).*

BMI group	SGA			LGA		
	Est	SE	*p*	Est	SE	*p*
Normal	0.856	0.018	0.001*	0.776	0.027	0.682
Overweight/Obese	0.925	0.013		0.790	0.022	

Supp. Table S7. EFW prediction results by BMI group for term pregnancy, for different birthweight categories, 14 days threshold. Continuous characteristics are presented as Mean±SD and compared between BMI groups using the nonparametric Wilcoxon rank sum test, categorical characteristics are presented as n (%) and compared between BMI groups using the Chi-square test (denotes significant difference between BMI groups).*

Birthweight Category	Characteristics	Term pregnancy		
		BMI		*p*
		Normal	Overweight/Obese	
SGA	n	59	34	
	EFW within 10% of birth weight	42 (71.2)	29 (85.3)	0.12
	EFW within 20% of birth weight	59 (100.0)	34 (100.0)	-
	Birthweight (g)	2676.0±235.5	2699.6±199	0.9
	GA at US visit (weeks)	38.2±1.1	38.3±1.2	0.3
	GA at delivery (weeks)	39.4±1.1	39.4±1.1	>0.9
	Time to delivery (days)	8.4±4.3	7.3±3.6	0.14
	Amniotic fluid index	11.7±3.8	11.7±4.0	0.999
	EFW (g)	2652.1±368.4	2649.5±255.1	>0.9
	Absolute error (g)	192.1±139.5	143.9±95.9	0.2
	Absolute percent error	7.2±5.1	5.5±3.9	0.2
AGA	n	564	434	
	EFW within 10% of birth weight	411 (72.9)	31 (71.7)	0.7
	EFW within 20% of birth weight	545 (96.6)	423 (97.5)	0.4
	Birthweight (g)	3370.6±301.0	3418.4±310.7	0.011*
	GA at US visit (weeks)	38.5±1.0	38.4±1.0	0.8
	GA at delivery (weeks)	39.6±1.0	39.5±1.0	0.6
	Time to delivery (days)	7.7±4.1	7.6±4.0	0.5
	Amniotic fluid index	14.1±4.7	14.3±4.7	0.6
	EFW (g)	3245.5±386.9	3307.5±410.6	0.027*
	Absolute error (g)	248.6±179.4	252.8±193.0	>0.9
	Absolute percent error	7.4±5.3	7.4±5.6	0.8
LGA	n	50	75	
	EFW within 10% of birth weight	35 (70.0)	49 (65.3)	0.6
	EFW within 20% of birth weight	49 (98.0)	72 (96.0)	0.6
	Birthweight (g)	4083.1±245.5	4183.3±265.6	0.015*
	GA at US visit (weeks)	38.3±0.9	38.3±1.0	0.8
	GA at delivery (weeks)	39.4±0.8	39.5±0.8	0.4
	Time to delivery (days)	7.9±4.0	8.1±4.2	0.8

Amniotic fluid index	16.9±4.9	16.1±5.3	0.3
EFW (g)	3832.5±384.6	3955.2±464.5	0.047*
Absolute error (g)	333.5±223.0	349.3±301.1	>0.9
Absolute percent error	8.2±5.4	8.4±7.2	0.8

Supp. Table S8. AUC table for nulliparous women, 14 days threshold (denotes significant difference between BMI groups based on the Wald test).*

BMI group	SGA			LGA		
	Est	SE	*p*	Est	SE	*p*
Normal	0.812	0.027	0.009*	0.779	0.042	0.655
Overweight/Obese	0.902	0.021		0.803	0.036	

Supp. Table S9. EFW prediction results by BMI group for nulliparous women, for different birthweight categories, 14 days threshold . Continuous characteristics are presented as Mean±SD and compared between BMI groups using the nonparametric Wilcoxon rank sum test, categorical characteristics are presented as n (%) and compared between BMI groups using the Chi-square test (denotes significant difference between BMI groups).*

Birthweight Category	Characteristics	Nulliparous pregnancy BMI Normal	Nulliparous pregnancy BMI Overweight/Obese	*p*
SGA	n	38	22	
	EFW within 10% of birth weight	27 (71.1)	21 (95.5)	0.041*
	EFW within 20% of birth weight	38 (100.0)	22 (100.0)	-
	Birthweight (g)	2599.2±309.5	2671.0±259.7	0.5
	GA at US visit (weeks)	37.8±1.3	38.1±1.5	0.2
	GA at delivery (weeks)	39.1±1.5	39.1±1.5	0.9
	Time to delivery (days)	8.7±4.4	6.9±3.4	0.068
	Amniotic fluid index	12.1±4.2	11.8±4.0	0.8
	EFW (g)	2590.5±459.4	2644±292.4	0.7
	Absolute error (g)	176.7±126.8	127.4±84.0	0.2
	Absolute percent error	6.8±4.7	4.9±3.3	0.15
AGA	n	300	165	
	EFW within 10% of birth weight	219 (73.0)	120 (72.7)	>0.9
	EFW within 20% of birth weight	292 (97.3)	159 (96.4)	0.6
	Birthweight (g)	3302.0±361.4	3329.6±428.0	0.3
	GA at US visit (weeks)	38.3±1.4	38.2±2.2	0.8
	GA at delivery (weeks)	39.4±1.4	39.3±2.1	>0.9
	Time to delivery (days)	7.7±4.1	8.0±4.2	0.5
	Amniotic fluid index	13.8±4.8	14.2±4.8	0.4
	EFW (g)	3182.0±429.5	3244.9±534.5	0.2
	Absolute error (g)	242.6±169.8	244.8±185.8	0.7
	Absolute percent error	7.4±5.1	7.5±5.7	0.8
LGA	n	21	25	
	EFW within 10% of birth weight	14 (66.7)	18 (72)	0.7
	EFW within 20% of birth weight	21 (100.0)	25 (100.0)	-

Birthweight (g)	3965.0±258.6	4197.4±201.6	0.001*
GA at US visit (weeks)	38.0±1.2	38.6±1.0	0.12
GA at delivery (weeks)	39.2±1.1	39.8±0.9	0.07
Time to delivery (days)	8.1±4.4	8.3±4.2	>0.9
Amniotic fluid index	15.4±5.1	15.9±4.9	0.5
EFW (g)	3751.3±421.9	4038.3±354.9	0.014*
Absolute error (g)	328.5±193.1	257.9±215.9	0.14
Absolute percent error	8.3±4.9	6.2±5.2	0.09
